# Spleen Histopathological Evaluation of Broiler Chickens Challenged with *Escherichia coli* and Its Effect Towards the Combination of Javanese Cardamom and Turmeric Herbs

**DOI:** 10.3390/vetsci12100975

**Published:** 2025-10-11

**Authors:** Tyagita Hartady, Mas Rizky A. A. Syamsunarno, Belgia Basyirasaniyanti, Shafia Khairani, Aziiz Mardanarian Rosdianto

**Affiliations:** 1 Department of Biomedical Science, Padjadjaran University, Jatinangor 45363, Indonesia; rizky@unpad.ac.id (M.R.A.A.S.); shafia@unpad.ac.id (S.K.); a.m.rosdianto@unpad.ac.id (A.M.R.); 2 Study Program of Veterinary Medicine, Padjadjaran University, Jatinangor 45363, Indonesia; belgia20001@unpad.ac.id

**Keywords:** 1,8-cineol, *Amomum compactum Sol. Ex Maton*, antimicrobial resistant, *Curcuma domestica Val*, colibacillosis, *Escherichia coli* O78, histopathology, spleen

## Abstract

**Simple Summary:**

Javanese cardamom and turmeric rhizome are Indonesian local herbs that contain antibacterial properties. As antibiotic resistance has become a growing concern, these herbs are commonly used as an alternative to antibiotics for treating bacterial infections. The objective of this study is to determine whether the combination of herbs can protect the chickens’ immune organs from damage caused by the disease. Researchers collected and analyzed the spleen tissue slides from infected chickens treated by three methods: (i) using a herbal combination, (ii) a single herb, and (iii) an antibiotic. Findings show that the chickens treated with the herbal combination had the least severe spleen damage. The combination acted as an antibacterial and a protective agent, effectively preserving the spleen’s structure and showing promise as an alternative treatment.

**Abstract:**

Given the increasing global concern over antimicrobial resistance in poultry health, this study investigated the potential of Javanese cardamom essential oil (JCEO) and dried turmeric (DT) as alternative therapies for colibacillosis by evaluating their effects on the spleen organ. A total of 72 Cobb-strain day-old chickens were allocated into eight groups, which received different doses of JCEO (0.06 mL/kg BW and 0.1 mL/kg BW), DT (400 mg/kg feed) and ciprofloxacin. Infection was induced intraperitoneally (*E. coli* O78 strain, 10^6^ CFU/mL/chicken) at 28 days, while the treatments were administered orally from day 7 to week 5. Histopathological evaluation was graded on a 1–5 scale based on the five primary lesion criteria. The herb combination groups had the lowest severity grade, characterized by compact lymphoid follicles and reduced vasculitis. The JCEO single-dose group, both in vitro and in vivo, reduced bacterial growth and had a mild vasculitis score, indicating its antibacterial activity. In contrast, the single treatment of DT and the antibiotic groups showed moderate spleen lesion damage. These findings suggest that JCEO acts bactericidally, while DT provides an anti-inflammatory effect, and both combinations work synergistically.

## 1. Introduction

Colibacillosis is a primary systemic pathologic disease caused by Avian Pathogenic *Escherichia coli* (APEC). The pathogen is often associated with several risk factors, including inadequate parental innate immunity, high stocking density, suboptimal ventilation, and poor sanitation. These conditions promote the horizontal transmission of APEC through fecal contamination and vertical transmission via eggs [1]. After entering the body, APEC spreads hematogenously and causes impairment of the bloodstream by its adhesion virulence factor. Each adhesion secreted by the bacteria is compatible with almost all receptors across chicken organs. After the adhesion process, the bacteria will secrete toxins to colonize the cell membrane. These toxins can disrupt cellular metabolism and lead to cell death.

The clinical signs typically appear between 3 and 5 weeks of age or progress as the agent infects. The disease often presents initially as respiratory tract and air sac infections (airsacculitis), which can progress to more localized infections and systemic infection [2]. Systemic infection is characterized by lethargy, anorexia, and dehydration [3,4]. The systemic manifestations are profound, causing significant disturbances to homeostasis, which leads to edema changes in specific organs (for example, hepatomegaly and splenomegaly) [5].

The spleen is a vital lymphoid organ associated with the sites of immune response activation following interaction with a pathogen. A unique physiological function of the spleen is its ability to mount both innate and adaptive immune responses, and it has been acknowledged that it can serve as a model organ to evaluate regulatory and catalytic mechanisms in drug design and inhibitor studies [6]. Throughout a chicken’s age, the modulation of immune function shifts following interactions with a pathogen. The spleen acts as a site where cell-mediated and humoral responses to antigens occur; thus, it warrants highlighting to assess pathological alteration responses to APEC virulence. Some studies indicate that damage to the spleen leads to immunosuppression. For this reason, the histopathological structure of the spleen can depict the progression of an APEC systemic infection. Microscopically, the damage originates from the vascular wall and extends through the infiltrated lymphoid tissue [7]. If the lymphoid structures are depleted, this can be an indicator of higher-severity infection in the chicken. Consequently, it increases susceptibility to other diseases [8].

Since colibacillosis is a highly pathogenic disease, it has caused substantial monetary losses, estimated at IDR 20 trillion. The significant loss is primarily due to lower carcass quality, reduced carcass yields, and increased culling rates [9]. To prevent this burden, local farmers commonly use antibiotic drugs because they are relatively affordable and accessible without a prescription, such as tetracyclines, sulfonamides, and aminoglycosides. A study in Sukabumi, Indonesia, reported that the *E. coli* isolated from clinical colibacillosis cases were resistant to oxytetracycline, enrofloxacin, ciprofloxacin, and tetracycline, with molecular analysis revealing that the resistance genes were highly increased [10]. Therefore, multi-drug resistance is an ongoingly studied issue in controlling colibacillosis.

In this urgent scenario, alternative treatments extracted from herbal plants have become available. Java cardamom (*Amomum compactum Sol. Ex Maton*) is a plant originating from Java Island, Indonesia. The seeds contain an active compound with antibacterial bioactivity and anti-inflammatory properties, specifically 2,4-dihydroxy-1,8-cineol. Another antibacterial compound from herbal plants is curcumin, which can be extracted with ethanol from turmeric (*Curcuma domestica Val*). In addition, turmeric also has anti-inflammatory and antioxidant properties which can protect cells against damage and promote cell longevity [5]. The effectiveness of curcumin in combination with other herbal antibiotic compounds for treating colibacillosis has been promising [11,12,13]. The main purpose of this study is to assess the effect of combining curcumin and 1,8-cineol on the broiler immune organ infected by APEC, which may yield better results than an approach using only one of either [14,15]. The urgent need to find alternative treatments for colibacillosis underscores the importance and relevance of our research.

## 2. Materials and Methods

While this study focuses on the histopathological analysis of broiler spleens, two primary steps had been previously conducted to collect the material for the experiment. First, we collected the herb extracts and the bacterial suspension, followed by constructing an in vitro study design. Secondly, we conducted in vivo experiments and a histopathological evaluation. The methods we used will be explained briefly below.

### 2.1. Herb Extractions

The Java cardamom and turmeric extracts were processed in accordance with a previous study [16]. The Java cardamom seeds were extracted with a steam-distilled process for six hours. The mixture of distilled oil was separated from water using a petroleum ether extraction method. The residual water was removed with anhydrous sodium sulfate until the final process of Javanese cardamom essential oil (JCEO) extraction was commenced, resulting in a density of approximately 0.98 g/mL.

For the turmeric rhizome, the study used a dried turmeric rhizome dissolved in 75% ethanol using a 500 mL Erlenmeyer flask at room temperature (22–26 °C) [17]. The turmeric powder and solvent were homogenized twice for five minutes and macerated for 48 h. The product was evaporated using a rotary vacuum evaporator at 60 °C to remove the ethanol. This extraction process allowed us to obtain 20.17 g of dried turmeric ethanol extract (DT). Both extracts were checked for their phytochemical compounds using gas chromatography–mass spectrometry and secondary metabolism components.

### 2.2. Bacterial Suspension and In Vitro Experiment

The *E. coli* O78 bacteria culture was ordered from the Center of Veterinary Diagnostic Laboratory in Bogor, Indonesia. The reasons for using this typical strain of *E. coli* were based on the common APEC serotypes found in broiler chickens. A study revealed that one of the most prevalent *E. coli* serotypes detected in broilers was O78. The bacteria also display notable resistance to penicillin and cefixime, but low resistance to norfloxacin and the quinolone group [18]. Confirmation of the presence of *E. coli* was performed using the Congo red dye agar test to differentiate between invasive and non-invasive *E. coli* strains in the experiment. After ensuring the presence of pathogenic *E. coli* strains, they were cultured in Nutrient Agar at 37 °C for 24 h.

To create the suspension, the bacterial culture was dissolved in a saline solution (0.9% NaCl) and then diluted in a nutrient broth medium to a final volume of 10 mL, resulting in a desired colony count of approximately 1–2 × 10^8^ CFU/mL [19]. Then, 100 μL of the suspension was added to the dilution medium (nutrient broth) until a final volume of 10 mL, resulting in a microbial suspension with a colony count of 1–2 × 10^6^ CFU/mL. After this, the bacterial suspension was used in an antibacterial test and an in vivo test.

Antibacterial activity testing was conducted in vitro using the microdilution method. This in vitro research method featured a completely randomized design consisting of five treatments with four repetitions, with the following samples being used in the antibacterial test:

A0 (negative control): Saline;
A1 (positive control): Ciprofloxacin;
A2: A combination of JCEO and DT;
A3: JCEO;
A4: DT.

The minimum inhibitory zone (MIZ) was measured using the disk diffusion method with concentrations of 100%, 50%, and 25% for each extract. Aseptically, *E. coli*, adjusted to the 0.5 McFarland standard, was swabbed on Nutrient Agar (NA) plates. Six-millimeter disks immersed in various herbal extracts were placed on the NA surface. The extracts were replicated three times for each dilution, using a complete randomized design. All agar plates were incubated at 37 °C for 24 h. The measured inhibition zone of the sample was compared with that of a positive control antibiotic (ciprofloxacin) which has a known sensitivity to *E. coli*. The diameter of the clear area, representing the zone of inhibition of the tested sample, was measured in millimeters, following the procedure used by Bauer et al. [20].

Meanwhile, the minimum inhibitory concentration (MIC) was determined by the serial dilution of antimicrobial agents from 32 or 64 μg/mL to 0.0625 μg/mL (i.e., 10 or 11 dilutions). Isolates from MacConkey agar were revived on NA (Oxoid Ltd., Dublin, Ireland) and cultured aerobically for 18–24 h before susceptibility testing was performed. The revived colonies were diluted to a 0.5 McFarland standard turbidity in 5 mL of demineralized water (Trek Diagnostic Systems Ltd., East Grinstead, UK). In total, 15 µL of the suspension was mixed in NA (Oxoid Ltd., Dublin, Ireland). Each well of the plate was inoculated with 50 µL of nutrient broth [21]. The plates were incubated at 37 °C for 24 and 48 h. The MIC for the isolates was determined from the well with the lowest concentration of antimicrobial agent, which produced no discernible growth of *E. coli*.

### 2.3. Animal Experiment Design

This study employed a completely randomized design (CRD) to investigate eight animal experiment design groups, as presented in Table 1. The calculation of the sample size was performed using Federer’s formula, which resulted in a minimum of three chickens per group, with three replications per treatment, resulting in a total of nine chickens per group. A total of 72 Cobb-strain, day-old, healthy broiler chickens (*Gallus gallus*) were used in the in vivo study. The study was conducted in the Animal Husbandry Laboratory of Padjadjaran University over a period of five weeks. The chicks weighed 920–930 g and were housed in separate cages in accordance with the experimental group design. The cage was built with galvanized steel and water-resistant wood. We ensured that each cage had adequate airflow with side ventilation. We set the cage temperature upon brooding recruitment for the first week (30–31 °C), and then reduced it by the next week until it reached 23 to 25 °C. The LED lamps provided lighting with an intensity of 20–30 lux. Filtered drinking water was supplied. Nutritional requirements (Hi-Pro-Vite 510 and Hi-Pro-Vite 511) for broilers, as outlined in the Cobb guidelines [22], were followed throughout the experimental period.

The bacterial suspension was injected intraperitoneally (IP) with an infectious dose of 10^6^ CFU/mL/chicken when the chickens were 28 days old, while the herbal extract was administered orally via drinking water from when they were 7 days old until 35 days old. The cage personnel were equipped with personal protective equipment and required to follow biosecurity protocols before entering the facility. Blinding was not performed during outcome assessment due to practical constraints in the experimental setup.

Clinical signs were observed daily for one week post-infection. At the end of the intervention period (week five), all chickens were slaughtered using the Halal Guidance technique. The necropsy for all experimental animals was performed in accordance with established protocols and conducted under biosafety level 2 (BSL-2) containment to ensure compliance with safety regulations. The organ samples were aseptically isolated using sterile specimen compartments soaked in a 10% formalin solution and stored in a container box, as described by Slaoui et al. (2017) [23].

### 2.4. Histopathology Preparation

The organ materials collected for the histopathology study included the intestine, lungs, heart, spleen, liver, bursa of Fabricius, and kidney. We sent the sample to Subang Veterinary Diagnostic Center for histopathological preparation. First, the organs were fixed in 10% buffered formalin for 24 h. The preparation was dehydrated with 70% ethanol for 2 h before being washed with toluene. The organs were embedded in paraffin wax, cut into 5 µm thick sections using a microtome, and placed on glass slides [23]. They were then stained with a hematoxylin and eosin stain. The stained tissue section was examined under a digital microscope, and scoring was performed using the ImageJ application.

### 2.5. Spleen Organ Scoring System

Five primary histopathological lesion descriptions in the spleen are explained in Table 2. Histopathological analysis was performed using a structured illumination microscope (Zeiss Apotome2, Jena, Germany) with magnifications of 10×, 60×, and 400×, and the ImageJ application (version 1.54d; NIH, Washington, DC, USA) to quantify the affected area (%). These tools helped to avoid observer bias and maintain consistency throughout the observation process. Each treatment group required 10 fields of view per sample to preserve consistent data between groups [24]. The affected area (%) was then graded on a scale of 0 to 5 to indicate the order of progression or change in severity [25,26]. A lesion nomenclature was used to aid qualitative description analysis (not marked, low, mild, moderate, severe, and highly severe), according to the following criteria presented in Table 3 [27]. The total lesion score is calculated by five severity scores (ranging from 0 to 5) and five lesion descriptions, resulting in a maximum score of 25.

The scoring distribution was assessed using the D’Agostino–Pearson normality test. Each group was significantly subjected to an ordinary one-way ANOVA statistic and multiple group comparisons by Tukey’s post hoc test. The results yielded a set of decisions, with *p* < 0.05 being considered statistically significant. All data and tables presented were obtained using GraphPad Prism for Windows, version 10.

## 3. Results

### 3.1. Data Analyses of Herbs’ Bioactive Compounds, MIC, and MIZ Results

The result of our analysis of the herbs’ bioactive compounds showed that the most abundance substance in JCEO is eucalyptol, also known as 1,8-cineole, with retention times of 15.7 min (area = 39.05%), 15.8 min (area = 2.87%), and 15.82 min (area = 20.92%). The quantitative analysis of DT revealed the presence of curcumin in an amount of 54.82% at a retention time of 2.30 min, supported by the molecular weight. The chromatogram results indicated the presence of (ar)-turmerone, curlone, and turmerone in DT. Phenols, tannins, steroids, and triterpenoids were also collected as phytochemical substances The results are attached in Supplementary Figures S4 and S5.

The assessment of antibacterial activity was conducted in the herbal group, at individual doses, and at combination doses. For the minimum inhibitory zone, all active concentrations of JCEO were able to show an inhibition zone in the bacteria’s growth compared to the negative control (DMSO). In contrast, DT extracts were ineffective in preventing the growth of the *E. coli* strain at any dose (see Supplementary Figure S6). The measurements of the antibacterial activity are shown in Supplementary Files Table S2. For the minimum inhibitory concentration, the results against *E. coli* suggested that a single dose of JCEO can exhibit bacteriostatic activity at an effective concentration of 1.563%. The DT did not show an inhibition zone against the tested bacteria. The combination of JCEO and DT exhibited antibacterial efficacy at a concentration of 6.25%. The combination of JCEO and DTEE exhibited antibacterial efficacy against *E. coli* O78 (12.5%), as indicated by the clarity of the well (results are attached in the Supplementary Files: Tables S3, S3-A and S3-B, and Figures S7 and S8).

### 3.2. Scoring Results

Every pathological lesion was calculated from ten views of each individual in the group, presented in Table 4.

The result for group C2 showed the highest score across all lesion parameters, indicating a severe and adverse effect of *E. coli* O78 on the spleen without any intervention. For the herbal combination group, C3 and C4 showed mild scores, especially since the necrosis and lymphoid depletion parameters were the lowest among the other groups. The standalone dose of JCEO in C5 and C6 did not show a significant difference in the statistical multi-comparison analysis (*p*-value > 0.999). In contrast, C7, which was preserved as a single dose of DT, yielded a severe number of pathological lesions compared to other herbal groups, but still maintained scores below those of the positive control group. Lastly, for the antibiotic group (C8), the score was lower than that for an individual dose of DT.

**Figure 1:**
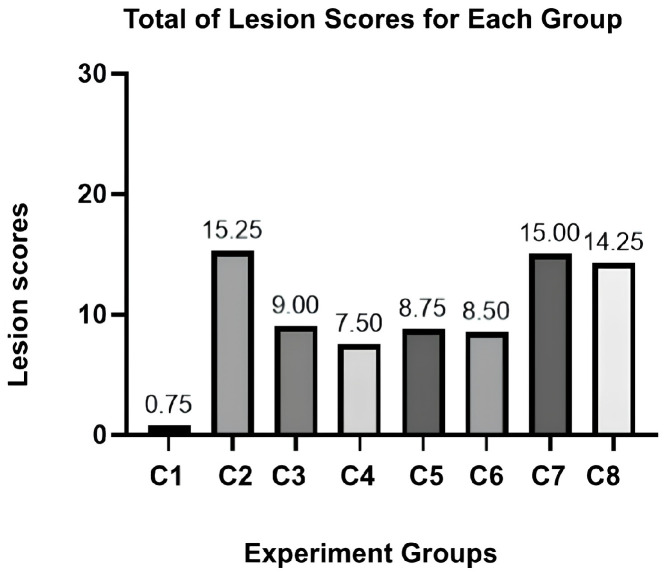
Total lesion scores for each experiment group.

The scores for vasculitis, necrosis, and lymphoid depletion were notably significant (*p*-value < 0.0001). At the same time, congestion and degeneration cells showed no significant results across the groups (see Supplementary Table S2). We also summarized the total lesions observed in Figure 1. The maximum number of lesions that could be acquired was 25 lesions per group. C2 was expected to be the highest lesion score, followed by scores for the C7 and C8 groups. While the parameters may have exhibited different tendencies between groups, it was possible to prove the presence of spleen damage microscopically.

### 3.3. Histopathology Assessment

A comparison was made between the broiler spleen structures of all groups at a low magnification (refer to Supplementary Figure S1 for detailed images). C1 proceeds as a standard broiler spleen structure, depicting a clear section between the red pulp (RP) and white pulp (WP), with a compact splenic artery (A) vessel wall. The WP is a combination of the central arteriole, splenic follicles, periarterial lymphoid sheath (PALS), and marginal zone. The structure is dense and basophilic, and appears more bleached with H&E stains because it contains active B cells and macrophage.

Meanwhile, the RP is collocated with parenchyme and venous sinuses, which act as an empty vascular space lined by endothelial cells. The sinus venosus functions to filter red blood cells (RBCs). The red pulp contains macrophages, granulocytes, plasma cells, mast cells, and RBCs and appears more reddish in the stain. This may be mistaken for a hemorrhage lesion (a bleeding out from the vascular wall) within the RP sinusoid. For a more discerning deduction, the lesion was then removed from the analysis indicator to ensure an unbiased judgment.

The experimental result, as shown in the C2 group (Figure S1B), indicates that the WP and RP structures appear coalescent. This is because the cells are undergoing degeneration so they cannot maintain their dense structure. The blood vessel was heavily affected, resulting in a narrowed lumen. The improvement was seen in the C3 and C4 groups. The integrity of the spleen structure is well visualized. In C5 and C6, a single dose of JCEO provides a better structure for WP and RP than in C7. All groups exhibited a moderate vasculitis score. For antibiotic treatment (Figure S1H), some features of the WP and splenic follicles -were visible, and based on the scoring, the severity of vascular damage was lower than that in C7.

## 4. Discussion

### 4.1. Systemic Route of APEC Infection of the Spleen and Its Effect on Vascularisation

**Figure 2:**
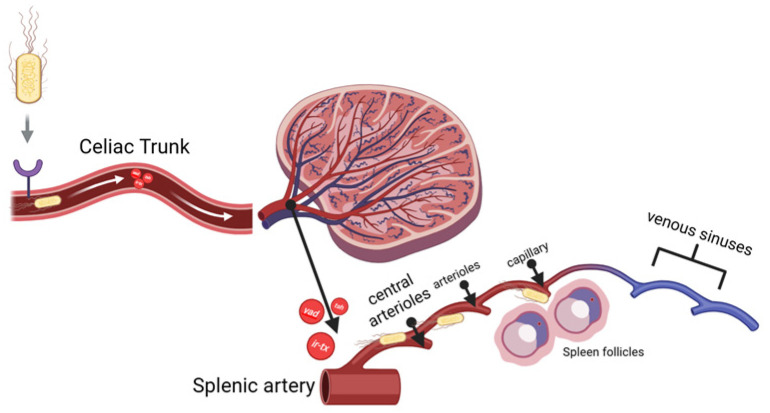
Bacterial sepsis through spleen vascularization branches.

As stated previously in the introduction section, the spleen’s anatomical structure favors APEC systemic infection due to its ability to adhere to the host. APEC was injected intraperitoneally, and it spread through the omental cavity and capillaries. The adhesins, for example *yad*, *ecp*, and *tsh*, and the iron-binding toxin find their receptors in endothelial cells and RBCs. As they attach, they can damage endothelial cells in blood vessels, leading to vacuolization and a loss of intercellular adhesion between cells. When the bacteria finally reach through the splenic artery (A), they branch into the central arteriole (CA), which divides into smaller vessels, including central arterioles, sheathed arterioles, and capillaries. These small blood vessels are located entirely within the WP and RP. The septicemia is simplified and visualized in Figure 2 below.

**Figure 3:**
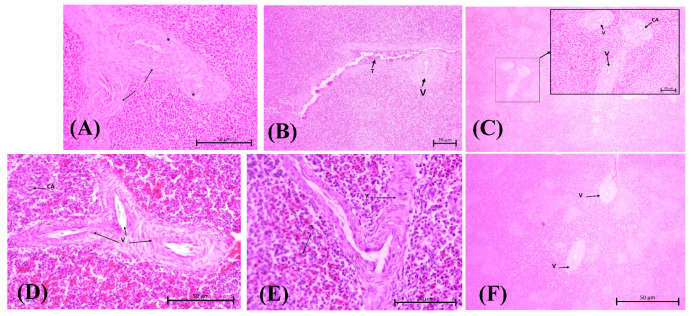
Vasculitis assessment for herbal group at medium magnification (100×, H&E stain) (**A**): Group C2; (**B**): Group C3; (**C**): Group C4; (**D**): Group C5; (**E**): Group C6; (**F**): Group C7. (**A**) Vasculitis (V) is observed along with the surrounding fibrin network (black arrow). There are also some vacuolated endothelial cells (black stars), and the lumen of the blood vessels has narrowed. (**B**) Vascular changes in C3 with central arterioles experience vasculitis and erythrocyte accumulation in the endothelium (T). (**C**) Group C4 vasculitis is indicated in the portal veins and central arteriole (CA). (**D**,**E**) Vasculitis causes different blood vessels to be adjacent to each other and narrows the lumen of the splenic cord (S). (**F**) Group C7 shows both damaged portal arteries (V); note the cell degeneration surrounding the blood vessels.

*Tsh* toxin is one of the by-products of APEC adhesion. It affects the degradation of the extracellular matrix (ECM), specifically collagen factor IV, resulting in damage to the vascular wall [30]. An *iron-binding toxin* is an endotoxin that binds to hemoglobin, causing erythrocyte lysis and allowing bacteria to escape from the bloodstream and spread within the inner structure of the spleen. This process occurs more quickly when a bacterial suspension is injected. These results trigger an immune response toward *E. coli* antigens, which is recognized via DAMP signal transduction to mast cells, macrophages and dendritic cells. Vascular permeability will increase to activate lymphocytes at the infection site. The infiltration of fibrinogen into the tissue causes a thickening of the tunica media and adventitia in blood vessels as an effort to replace the damaged endothelial cells [31]. Congestion also results from the lysis of blood cells and the infiltration of macrophages. Lesions are well described for the C2, C5, and C7 groups (Figure 3).

The results of the ANOVA test on vasculitis parameters were highly significant (*p* < 0.0001, refers to Supplementary Table S1). The C2 score was the highest vasculitis score, at 4.250, among all other test groups, as the purpose of mimicking the *E. coli* pathogenic systemic route was achieved as intended. In Figure 3B, vasculitis is depicted by fibrosis and a thickened tunica adventitia wall, which causes it to coalesce with other vessels (V). Higher magnification reveals the surrounding fibrin network (indicated by the black arrow), vacuolated endothelial cell (black stars), and lumen with narrowed blood vessels. The vasculitis condition was observed to be better in the C3, C4, and C6 groups, as indicated by a less thickened wall. There are no significant differences between the herbal groups. However, C7 showed more pronounced progression of vascular damage.

The issue with C7 is the metabolic process of curcumin, which involves several steps of digestion and the bioactivity attribute itself. First, curcumin’s mechanism of action is the disruption of biofilm formation and induction of prolonged bacterial death, whereas 1,8-cineol exhibits a more direct and bactericidal mechanism against bacterial cells. Our in vitro findings showed that the absence of an inhibition zone indicated that using DT in a single dose did not affect the tested bacteria (see Supplementary Figure S6 and Table S1). Several studies have found that curcumin’s limited antibacterial activity is attributed to its unstable chemical structure, resulting in decreased pharmacokinetic and pharmacodynamic properties [32].

Secondly, the drug formulation determines the metabolic process of curcumin. Curcumin emulsion (oil-in-water) formulations are better absorbed than oil-based formulations and maintain curcumin stability in the body even under neutral and alkaline pH conditions [32]. Its natural state is unstable, and it degrades into other components such as vanillin, which reduces the pharmacodynamic half-life. In the future, a more effective formulation may be developed to enhance its mechanism efficiently.

The reason for vascular lesions being observed across all groups was because the oral administration of herbs and antibiotics is associated with a long pharmacokinetic window before they reach the target organ. Meanwhile, APEC spreads more rapidly through hematogenous infection following peritoneal injection. Vasculitis parameter results can confirm that bacterial infections were indeed progressing systematically by destroying the vascular cell walls of the spleen.

### 4.2. APEC Toxins’ Effect on the Spleen’s Inner Structure

We now understand how APEC can easily penetrate the spleen structure. To assess the severity of infection, the spleen’s inner structure must be observed thoroughly. Pathologically, cells can undergo two different states of injury when exposed to antigens. First is a non-lethal injury known as cell degeneration. Cell degeneration is a pathological condition that causes changes in the structure of the cell’s cytoplasm. This injury results in the abnormal accumulation of material in the cytoplasm [31]. The cell might be reversibly improved if the pathological stimulus is reduced or other substances are added that reduce cell damage, such as antioxidants and anti-inflammatory agents.

The other injury is cell necrosis. Necrosis refers to the changes that occur after cell death or irreversible damage. Necrotic changes in the spleen are characterized by swelling of cell organelles, changes in the nucleus leading to an accumulation of nuclear debris in the cytoplasm, eosinophilic karyohecticity, and abundant karyolysis. Additionally, the cell may also be accompanied by inflammatory cell infiltration, hemorrhage, and a loss of the cytoplasmic membrane [33].

These types of pathological changes were found to vary in APEC infections. The mechanism of cell degeneration is triggered by the APEC *Stx2* toxin (verotoxin-2), subA-B toxin, and VAT [29]. SubA has catalytic activity and is responsible for the cytotoxic effect. SubB binds to receptors on the cell membrane surface, facilitating the entry of toxins into the cell. VAT alters the cell cytoskeleton and decreases epithelial permeability. Both toxins induce cell vacuolization. This results in the movement of the extracellular matrix into the cell, causing the cell to swell like a cloud and the cytoplasm to appear pale/opaque in H&E staining. Another virulence factor of APEC includes hemolysin, chick-lethal toxin, and CNF. This virulence is responsible for necrotic spleen cells [29].

Histopathological results illustrate the difference between the various groups of spleen structures (see Supplementary Figure S2). The negative control groups (C1) exhibited the typical structure of the chicken spleen. The WP was dense, characterized by lymphoid follicles containing lymphocyte cells with intact marginal zones. The central arteriole should have been inside the PALS, which was surrounded by mature T cells. This is crucial since its function is for lymphocyte cell maturation and the formation of antibodies.

The RP zone needs to be able to identify the two constituent components: the splenic cord of Bill Roth and the sinus venosus. It is characterized by an empty, flattened cavity, with the tip fused to the other venous sinuses. Damaged RBCs do not pass through the sinusoidal filtration and accumulate, eventually being ingested by macrophages circulating in the spleen.

Statistically, there were no significant differences between the treatment groups and the positive control group in terms of cell degeneration (*p*-value > 0.05). However, the observation suggests that these cells had changed. In infected groups, the visible alterations included vacuolar degeneration and cytoplasmic degeneration. The cells appeared cloud-like due to the influx of ions, plasma fluid, and water into the cytoplasmic matrix, causing the cells to expand and swell, producing a cloudy, central stain known as ghost cells (Figure S2).

Meanwhile, for necrosis, there was a significant difference in the affected area between each of the treatment groups. The most severe necrosis condition was shown in C7 and C8, with necrosis scores of 3 ± 1.826 and 4 ± 0.816, respectively. The comparison of necrosis conditions between the C8 group and both herbal groups was highly significant (*p* value = 0.0007). The WP cells, especially the lymphocyte nuclei in C7 and C8, were observed with karyohexis. This can be seen from the loss of the nucleus and also of the membrane of the spleen cell wall, causing the cells, which are colored pale pink (blue stars), to appear fused in the cytoplasm.

When there are too many injuries, necrotic cells can cause follicle depletion. This condition involves injury to lymphocyte cells, leading to a decrease in the size of the lymphoid follicle within the white pulp. It can present a few or no germinal centers, a complete loss of the marginal zone, and irregularity of the follicle shape [34]. If the severity increases, the zone between WP and RP will be coalescent and indistinguishable.

All herbal treatment groups showed insignificant differences in necrosis scores compared to the negative control group. This indicates that the necrosis condition of the spleen organ in the herbal group was relatively similar to the normal histology of the broiler spleen. Assessing lymphocyte cell injuries is essential because this allows us to interpret the density of the lymphoid follicle in the WP, thereby promoting long-term immune defense in the chicken.

C1 (see Figure S2A–C) presents references to detailed features in the WP and RP structure. The key difference in the C2 group is the presence of necrotic lymphocytes within the follicles, as well as in the PALS zone and RP (Figure S2D). Cells in the PALS zone and red pulp exhibited morphological changes, with fluid accumulating in the cytoplasm and pushing against organelles within the cells, resulting in swelling. In the herbal group, C3 and C4 had the best follicle condition compared to the other groups, with scores under <1% for the area affected. These can be seen by the integrity of the marginal zone, which is well separated into WP and RP.

In contrast, the morphology in C5, C6, and C7 (see Figure S2E–G) shows that the marginal line separating the red pulp and white pulp is no longer visible. The follicles are filled with nuclear debris and cell cytoplasm, indicating that the lymphocyte cells are experiencing necrosis and degeneration. The central arteriole in the group exhibits cell wall damage and tunica thickening, even though the difference is considered insignificant. For the antibiotic groups, the results are similar to those of the negative control. The follicle is visible with several necrotic lymphoid cells around it.

The significant differences in lymphoid depletion in the herbal groups and antibiotic group suggest that the cell improvement might be from 1,8-cineol and curcumin effects. The substance proactively inhibits the bacterial virulence, at the same time modulating immune cells to be more resistant against injury. Each mechanism is widely known, yet the contributions to immune organs are still to be explored.

### 4.3. Roles and Mechanisms of JCEO and DT in Protecting Spleen Cells

It has been demonstrated that the bioactive compounds in JCEO lead to the production of 1,8-cineol. The in vitro results also showed the JCEO optimum dose result for inhibiting bacterial growth (please refer to the additional information in Supplementary Figures S6–S8 and Table S3, S3-A and S3-B). Cineol has two mechanisms of antibacterial activity. The first mechanism is the disruption and alteration of the structure of the *E. coli* biofilm being formed, which consists of an extracellular polymer matrix [35]. The initial mechanism involves binding to porin (a transmembrane protein) on the outer membrane of the bacterial cell wall. This bond results in damage to the porin, which reduces the permeability of the bacterial cell wall, ultimately leading to bacterial death due to changes in the hydrophobicity of the *E. coli* membrane. This is what makes lipopolysaccharides in *E. coli* more susceptible to cineole [5].

The second mechanism involves disrupting a quorum-sensing system in *E. coli*, specifically the *LuxS* protein from the *autoinducer-2* (*AI-2*) system [30]. This process results in bacteria that cannot express the *LuxS* protein; therefore, they lack adhesion genes, which are considered virulence factors, as we have discussed before. In addition to its antibacterial activity, some studies have explored the anti-inflammatory properties of cineol. They have shown that cineole can reduce inflammatory cytokines and reactive oxygen radicals (ROS) by inhibiting cyclooxygenase 2 (COX-2) production (refer to Supplementary Figure S3A) [35,36].

Curcumin has been investigated for its potential to genetically enhance growth in broiler chickens, reduce oxidative stress, and stimulate the formation of immune cells (Figure S3B). Studies on high-stock-density chickens have shown that curcumin can significantly increase the synthesis of humoral immunity, by escalating the proliferation of B and T lymphocytes produced in lymphoid follicles. Therefore, the spleen can efficiently produce plasma cells, which then secrete antibodies [37,38].

**Figure 4:**
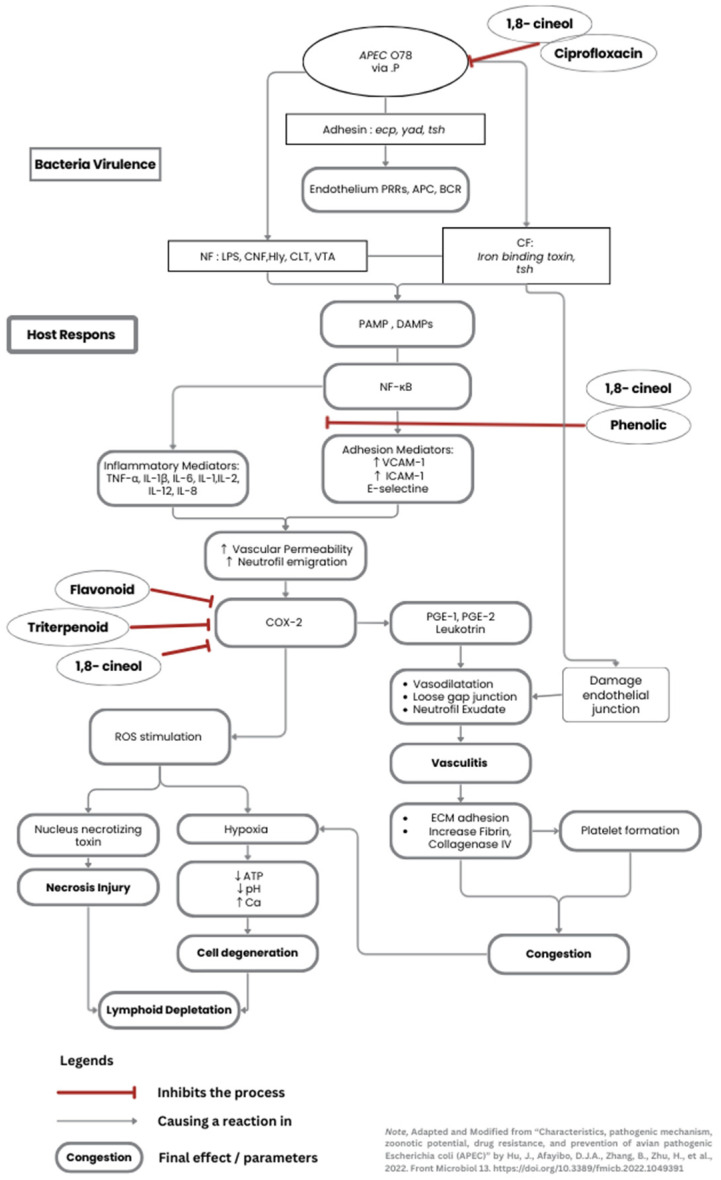
The pathogenicity of systemic APEC affects the cells (Adapted and modified from Hu et al. [28]). The diagram shows the main bacterial virulence alongside the host immune response. An additional pathway involving ciprofloxacin, JCEO, and DT substances is briefly mentioned, which inhibits specific infection processes. (1,8-cineol is the primary substance for JCEO. Phenol, flavonoids, triterpenoids are found in the DT extraction end-results).

Overall, these properties work well in combination, even though the antibacterial activity was shown to have reduced. Primarily, JCEO has a potent antibacterial effect, and DT can generally support immunomodulation. The antibacterial activity of 1,8-cineol is attributed to the disruption of bacterial cell wall permeability, thereby preventing the bacteria from penetrating deeper into the splenic structure. Even though some necrotic cells were visible around the blood vessels due to the APEC endotoxin effects, cineol also had an anti-inflammatory effect, minimizing additional cell injuries. Simultaneously, DT enhanced the differentiation of lymphocytes and reduced the production of inflammatory cytokines. When it is given as a routine supplement, the immune organ already has an adequate and mature environment for immune cells to reduce injuries at the moment the chicken is infected. The authors created a diagram illustrating the pathogenicity of APEC, as well as the mechanisms by which cineol and curcumin affect spleen cells, shown in Figure 4.

### 4.4. The Effect on Other Organs

Two similar studies conducted the same experiment with different organ analyses. The herbs’ and antibiotics’ effects were observed in broiler hearts and lungs challenged with the same APEC suspension [39,40]. Vasculitis, thrombus, and granulation tissue were the highlighted pathological findings in the heart. Severity scores in the lungs appeared insignificant, but typical pathological lesions were found in all groups (pneumocyte inflammation and abnormality of the parabronchi structure). The total plate count (TPC) data from the intestinal tract was collected to support the determination of the antibacterial activity of the herbs (details in Supplementary Table S4). Overall, the lesions were similar between organs. The damage to the vascular wall and infiltration of granulocytes within the cells indicated the effects of adhesion and the endotoxin from APEC.

An interesting discovery regarding dosages was made; specifically, the combination of a 0.06 mL/kg BW JCEO dose with the 400 mg/kg feed of curcumin showed greater improvement in the lungs. In contrast to the heart and spleen, a higher dose of JCEO was more effective in protecting cells. The study concludes that higher doses of cineol may cause side effects on the digestive tract, resulting in a high feed consumption rate due to intestinal damage [39]. There is insufficient evidence to support this argument, as it does not demonstrate how 0.1 mL/kg BW JCEO resulted in a better outcome in well-vascularized organs.

Both studies reported that antibiotics resulted in a moderate severity score. At this point, the same scenario also happened in the spleen, even though the experiment adhered to the manufacturer’s standardized antibiotic dosage. A degradation in antibiotic efficacy is likely to occur when antibiotics are administered through drinking water. The powdered dosage formulation exhibited slower pharmacodynamic activity than the injectable dosage form, whereas the APEC was administered peritoneally. Furthermore, the chickens received no additional supplements or special care during the rearing period. The chickens’ immune systems may have been weakened compared to those in other groups treated with herbal treatments that have immunomodulatory effects. The antibiotic group did not exhibit additional anti-inflammatory properties. As a consequence, the depletion of follicles was irreversible.

## 5. Conclusions

The conclusions drawn in the discussion section were based on the histopathological assessment of the spleen organ, despite some limitations of the study. The lowest spleen lesion histopathology scores were observed in the combination group, which consisted of JCEO 0.1 mg/kg BW and DT 400mg/kg feed. The antibacterial activity of 1,8-cineol and the anti-inflammatory effect of curcumin can be observed through the spleen’s inner structure, which revealed the compact density of lymphoid follicles, slight condition vasculitis, and a distinct structure of WP and RP. The histopathological features observed also revealed these outcomes.

Instead of H&E staining, using more precise staining methods, such as immunochemistry, may improve the differentiation of specific lymphocyte cells. Masson Trichrome and silver staining can also be used to easily analyze the fibrin matrix structure for vascular damage. If the bacterial debris could be seen within the cells, the evidence would be more convincing. Other vital organs, especially the bursa of Fabricius, need to be assessed immediately to obtain comprehensive evidence.

The broiler spleen scoring system still requires standardization due to research in this specific field being scarce. This study modified the pathology assessment based on a practical case of spleen injuries caused by the *Escherichia coli* pathogenic strain [41]. Other exposures to injuries, besides those caused by bacteria, may result in different lesions to be assessed.

Therefore, for the fundamental analysis of spleen histopathology, this study recommends using the enhanced histopathology of the spleen and the practice guidelines for the pathological evaluation of the immune system [41,42]. By understanding the basic compartment and histological structure of the spleen, the characteristics of any other agent can be thoroughly analyzed.

## Figures and Tables

**Table 1:** In vivo animal experiment design groups.

Groups	Treatments
C1	Received an oral 0.5 mL saline solution only (0.9% NaCl) without any infection or treatment
C2	Challenged with 0.5 mL *E. coli* O78 suspension IP and received an oral 0.5 mL saline solution only (0.9% NaCl)
C3	Challenged with 0.5 mL *E. coli* O78 suspension IP and received 0.06 mL/kg BW of JCEO + 400 mg/kg feed/day of DT orally.
C4	Challenged with 0.5 mL *E. coli* O78 suspension IP and received 0.1 mL/kg BW of JCEO + 400 mg/kg feed/day of DT orally.
C5	Challenged with 0.5 mL *E. coli* O78 suspension IP and received 0.06 mL/kg BW of JCEO orally
C6	Challenged with 0.5 mL *E. coli* O78 suspension IP and received 0.1 mL/kg BW of JCEO orally
C7	Challenged with 0.5 mL *E. coli* O78 suspension IP and received 400 mg/kg feed/day of DT orally.
C8 *	Challenged with 0.5 mL *E. coli* O78 suspension IP and received Ciprofloxacin (10 mg/kg BW in 1g/2 L water) orally.

* C8: A group of chickens that received antibiotic treatment after five days of being infected.

**Table 2:** Five primary histopathological lesion assessments.

Type of Lesions	Descriptions	Correlation to APEC Pathogenesis (Modified from [28,29])
Congestion	A blood clot, infiltration of a macrophage, or the presence of any bacterial-stained colonies is found inside the blood vessels. The vessel wall remains intact.	The bacteria may already be present systemically, but not potent enough to damage the spleen’s inner structure.
Vasculitis	Blood vessel inflammation. This can be in the primary portal or the capillary portal. The vessel wall becomes thickened and disorganized due to the presence of a fibrin matrix.	The bacteria adhered to the vessel cell wall, releasing an endotoxin that caused cell membrane disruption and damage, resulting in an immune response to repair the wall
Cell degeneration	Cells such as those in the vessel epithelium and lymphoid cells undergo a disruption. The nucleus is still present. White pulp remains well structured.	If vasculitis occurs, lymphoid cells would most likely be affected by endotoxin and CNF released from the bacteria.
Necrosis of lymphoid	The cell’s nucleus is either absent or undergoing karyohexis, karyolysis, or pyknosis.	The vasculitides are severe, and bacterial endotoxins are potent in damaging cells.
Lymphoid depletion	The overall structure of the white pulp is disintegrated, smaller, and more abstract due to the presence of necrotic lymphoid cells.	The vessel wall is severely infected. Thus, bacteria colonize the spleen’s inner structure

**Table 3:** Modified spleen lesion score for APEC-induced lesions.

Score	Area Affected	Severity
0	Normal	Not marked
1	<1%	Low
2	1–20%	Mild
3	21–60%	Moderate
4	61–80%	Severe
5	>81%	Highly severe

**Table 4:** Experimental group scoring lesions.

Groups ^1^	Histopathological Parameters Severity Grade (Mean ± SD) *				
	Vasculitis	Congestion	Cell Degeneration	Necrosis	Lymphoid Depletion
C1	1.25 ± 0.5	1.00 ± 0.577	1.50 ± 0	1.00 ± 0	1.00 ± 0
C2	4.25 ± 0.5	1.50 ± 0.577	2.5 ± 1.291	3.00 ± 0.816	3.75 ± 0.500
C3	2.75 ± 0.5	2.75 ± 0.5	2.5 ± 0.577	1.25 ± 0.5	1.75 ± 0.5
C4	2 ± 0.8165	0.75 ± 0.5	2 ± 0.8165	1.75 ± 0.957	1.75 ± 0.60
C5	3.25 ± 0.5	1.25 ± 0.957	1.75 ± 0.5	1.5 ± 0.557	1.5 ± 0.557
C6	2.25 ± 0.5	1.25 ± 0.5	1.75 ± 1.708	1.5 ± 0.577	1.5 ± 0.577
C7	4 ± 0.816	1.25 ± 0.5	2.75 ± 0.957	3 ± 1.826	2.5 ± 0.577
C8	3 ± 0.816	1 ± 0.816	2 ± 0.816	4 ± 0.816	3.75 ± 0.5

^1^ The group description referenced in Table 1. In vivo animal experiment design. * Mean ± SD: the average severity of lesions ± standard deviation, because the scores are accumulated from three replications in each experiment group.

## Data Availability

The original contributions presented in this study are included in the article/Supplementary Material. Further inquiries can be directed to the corresponding author.

## Supplementary Materials

The following supporting information can be downloaded at https://www.mdpi.com/article/10.3390/vetsci12100975/s1: Figure S1: An overview of the histopathological assessment in the broiler spleen experimental group at a low magnification (20×, H&E Stain); Figure S2: Spleen WP and RP architecture damage across the groups at a high magnification (400×, H&E Stain); Figure S3: The mechanism of cineol against bacteria (A) [37] and curcumin’s properties (B) towards immune response [43]. Figure S4: Physical properties and gas chromatography–mass spectrometry results for Javanese cardamom essential oil (JCEO); Figure S5: Physical properties, phytochemical test, and gas chromatography–mass spectrometry results for dried turmeric ethanol extract (DT). Figure S6: Antibacterial activity of extracts against the *E. coli* O78 strain; Figure S7: MIC of JCEO:DT combination (1:1) against *E. coli* O78; Figure S8: MIC of JCEO against *E. coli* O78. Table S1: ANOVA Multiple comparisons statistical data on each parameter; Table S2: Antibacterial activity of JCEO and DT against *E. coli* O78; Table S3. MIC of JCEO against the tested *E. coli* O78 strain; Table S3-A. MIC of JCEO against *E. coli* O78; Table S3-B. MIC of JCEO and DT combination (1:1) against *E. coli* O78; Table S4. Mean of total plate count (TPC) of intestinal tract isolation oneweek post-infection [44].

## Abbreviations

The following abbreviations are used in this manuscript:

A	artery or splenic artery
ANOVA	Analysis of Variance
APEC	Avian Pathogenic *Escherichia coli*
BW	body weight
°C	Celsius
CA	central arteriole
COX-2	cyclooxygenase 2
CNF	cytotoxic necrotizing factors
CFU	colony-forming unit
DAMPs	Damage-Associated Molecular Patterns
DT	dried turmeric ethanol extract
*ecp*	Escherichia common pilli
ECM	extracellular matrix
F	follicle or splenic follicle
g	gram
Irb-toxin	iron-binding toxin
H&E	hematoxylin and eosin
IP	intraperitoneal
JCEO	Javanese cardamom essential oil
MIC	minimum inhibitory concentration
MIZ	minimum inhibitory zone
mL/kg	milliliter per kilogram
mg/kg	milligram per kilogram
NaCl	natrium chloride
PALS	periarterial lymphoid sheath
RBC	red blood cells
ROS	reactive oxygen species radicals
RP	red pulp
TPC	total plate count
*tsh*	temperature-sensitive haemagglutinin
*yad*	Yad fimbriae
V	vasculitis
VAT	vacuolating autotransporter toxin
WP	white pulp
