# Supplementary material for: Spleen Histopathological Evaluation of Broiler Chickens Challenged with Escherichia coli and Its Effect Towards the Combination of Javanese Cardamom and Turmeric Herbs
Source: Vet Sci. 2025 Oct 11;12(10):975. doi: 10.3390/vetsci12100975 (PMC12568280; doi:10.3390/vetsci12100975)
Supplement: Supplementary file 1 [file vetsci-12-00975-s001.zip › vetsci-3878421-supplementary.pdf]

Supplementary Materials

Spleen Histopathological Evaluation of Broiler Chickens Challenged with *Escherichia coli* and Its Effect towards the Combination of Javanese Cardamom and Turmeric Herbs.

Tyagita Hartady [1,2,*], Mas Rizky A.A. Syamsunarno[1] , Belgia Basyirasaniyanti[2] , Shafia Khairani [1,2], Aziiz Mardanarian Roosdianto[1,2]

[1] Department of Biomedical Science, Padjadjaran University, Jatinangor 45363, Indonesia; rizky@unpad.ac.id

[2] Study Program of Veterinary Medicine, Padjadjaran University, Jatinangor 45363, Indonesia belgia20001@unpad.ac.id (B.B.); shafia@unpad.ac.id (S.K.); a.m.rosdianto@unpad.ac.id (A.M.R.)

*Correspondence author: tyagita@unpad.ac.id

Table S1. ANOVA Multiple Comparisons Statistical Data on Each Parameter

ANOVA summary	Vasculitis	Congestion	Cell Degeneration	Necrosis	Lymphoid Depletion
**F**	15,73	1,132	3,886	8,791	31,36
**P-value**	<0,0001	3,766	58	<0,0001	<0,0001
**P-value summary**	****	ns	**	****	****
**Significant diff. among means (P < 0.05)?**	Yes	No	Yes	Yes	Yes
**R squared**	821	2,482	5,313	7,194	9,014

Table S2. Antibacterial activity of Java cardamom essential oil (JCEO) and dried turmeric ethanolic extract (DT) against *E. coli O78* (Mean + SEM) (mm).

			***E. coli* O78**	
**No.**	**Sample**	**Concentration**	**(Mean+SEM) (mm)**	**Status**
**1**	JCEO	1.000 mg/ml	19.10±1.1	**Active**
	JCEO	500 mg/ml	12.18+0.5	**Active**
	JCEO	250 mg/ml	9.8±0.5	**Active**
	Ciprofloxacin	1000 ppm	36.0±0.3	**Active**
	DMSO	10 %	6.00±0	Not Active
**2**	DTEE	1.000 mg/ml	6.00±0	Not Active
	DTEE	500 mg/ml	6.00±0	Not Active
	DTEE	250 mg/ml	6.00±0	Not Active
	Ciprofloxacin	1000 ppm	32.9±0.6	Not Active
	DMSO	10 %	6.00±0	Not Active
**3**	JCEO: DTEE (1:1)	1.000 mg/ml	8.2±0.2	**Active**
	JCEO: DTEE (1:1)	500 mg/ml	7.2±0.2	**Active**
	JCEO: DTEE (1:1)	250 mg/ml	6.00±0	Not Active
	Ciprofloxacin	1000 ppm	33.2±0.4	**Active**

DMSO	10 %	6.00±0	Not Active

Table S3. MIC of JCEO against the tested *E. coli* O78 strain.

**Extracts**	***E. coli* O78**
	**Concentration (%)**
**JCEO**	1.563
**JCEO : DTEE (1:1)**	6.25
**DTEE**	Not applicable

Table S3-A. MIC of JCEO against *E. coli* O78. According to calculations, JCEO exhibits bacteriostatic activity against *E. coli* O78 at a minimum concentration of 1.563 %. While the solvent (DMSO 10 %) has no bactericidal action against *E. coli* O78.

**Well**	**Concentration (%)**											
	**25**	**12.5**	**6.250**	**3.125**	**1.563**	**0.781**	**0.391**	**0.195**	**0.098**	**0.049**	**0.024**	**0.012**
**Media + Sample**	0.164	0.070	0.063	0.055	0.047	0.045	0.045	0.045	0.045	0.044	0.044	0.044
**Media + Solvent**	0.040	0.042	0.043	0.043	0.044	0.044	0.044	0.045	0.045	0.044	0.044	0.043
**Media + Sample+ Bacteria**	0.140	0.094	0.093	0.076	0.062	0.083	0.204	0.396	0.396	0.410	0.469	0.391
**Media + Solvent + Bacteria**	0.319	0.310	0.460	0.556	0.535	0.557	0.557	0.611	0.611	0.587	0.653	0.650
**Cell death percentage**	**108.604**	**90.921**	**92.750**	**95.940**	**96.903**	**92.633**	**69.181**	**36.033**	**37.964**	**32.732**	**30.204**	**42.916**

Table S3-B. MIC of JCEO and DT combination (1:1) against *E. coli* O78. JCEO and DT (1:1) demonstrate promising bacteriostatic activity against *E. coli* O78 at a minimum concentration of 6.25%, according to the calculations. This finding instills hope for the potential of herbal combinations in inhibiting bacterial growth, while the solvent (DMSO 10 %) shows no bactericidal action against *E. coli* O78.

**Well**	**Concentration (%)**											
	**50**	**25**	**12.5**	**6.250**	**3.125**	**1.563**	**0.781**	**0.391**	**0.195**	**0.098**	**0.049**	**0.024**
**Media + Sample**	0.607	1.170	0.772	0.386	0.276	0.221	0.162	0.119	0.119	0.085	0.077	0.075
**Media + Solvent**	0.039	0.041	0.041	0.041	0.041	0.042	0.041	0.041	0.041	0.042	0.042	0.042
**Media + Sample + Bacteria**	0.439	0.641	0.538	0.486	0.456	0.424	0.311	0.342	0.354	0.313	0.341	0.356
**Media + Solvent + Bacteria**	0.163	0.256	0.308	0.330	0.351	0.390	0.416	0.423	0.452	0.467	0.485	0.543
**Cell death percentage**	**234.736**	**345.191**	**187.362**	**65.438**	**41.804**	**41.822**	**60.264**	**41.567**	**42.664**	**46.069**	**40.298**	**44.005**

Groups	Treatments	Mean ± SD
C1 (Negative control)	Received an oral 0.5 ml saline solution only (0.9% NaCl) without any infection or treatment	98,4 ± 55,7
C2 (Positive control)	*E. coli* O78 suspension IP + 0.5 ml saline solution only (0.9% NaCl)	272,6 ± 118,3
C3	*E. coli* O78 suspension IP + oral 0.06 ml/kg BW of JCEO + 400 mg/kg feed/day of DT.	47,3 ± 31
C4	*E. coli* O78 suspension IP + oral 0.1 ml/kg BW of JCEO + 400 mg/kg feed/day of DT.	14 ± 7
C5	*E. coli* O78 suspension IP + an oral 0.06 ml/kg BW of JCEO	7,5 ± 2,9
C6	*E. coli* O78 suspension IP + oral 0.1 ml/kg BW of JCEO	112,8 ±3,9*
C7	*E. coli* O78 suspension IP + oral 400 mg/kg feed/day of DT.	696 ±60
C8	*E. coli* O78 suspension IP + Ciprofloxacin (10 mg/kg BW in 1g/ 2 L water).	115,8 ± 51

Table S4. Mean of Total Plate Count (TPC) of intestinal tract isolation one-week post-infection.

Discussion:
From the TPC count, a significant difference was observed in C7, with 695 CFU/mL. A single dose of JCEO (C5) shared the lowest score (7.5 CFU/ml). Ciprofloxacin sensitivity (115,8 CFU/mL) was performed poorly compared to the herbal combination group. The conclusion drawn from this finding is that the DT single dose does not exhibit antibacterial activity. In groups C3 to C5, the chickens were given a mixture of both extracts from the start of their care. As a result, *E. coli* growth was inhibited, and there was no significant difference in results between the three groups on day 7 post-infection. This result is in line with the in vitro condition that the curcumin extract from the DT is not potent enough to inhibit *E. coli*. It then concluded that the leading antibacterial agent in this herbal combination is JCEO. All the bacteria from the TPC test were confirmed on Congo Red media. They demonstrated growth, indicating that pathogenic *E. coli* bacteria were indeed responsible for the infection in the chickens.

* A single high dose of JCEO resulted in higher TPC scores, as well as a moderate lesion score in the serosa and congestion. These findings are also supported by the low average body weight of the chicken in group C6 (5.328 grams). The incidence of colibacillosis in group 6 can be explained by the fact that a single, higher dose of JCEO was insufficient to protect the chickens from the virulence of the infected *E. coli.* The pungent aroma and heat of eucalyptol are suspected of irritating the digestive tract [44]. thereby suppressing the chickens' immunity and making it easier for *E. coli* to spread more easily in the chickens' bodies when infected simultaneously. This situation highlights the potential risks associated with the use of high doses of JCEO, particularly in cases of simultaneous infection. Conversely, this situation does not apply to other organs such as the heart, lungs, and spleen. All of the organs showed insignificant lesion damage. This is probably because the intestine is the first barrier to contact with eucalyptol, thus the heat effect is immediate. Meanwhile, the other organ has presumably already received the eucalyptol in reduced concentration.

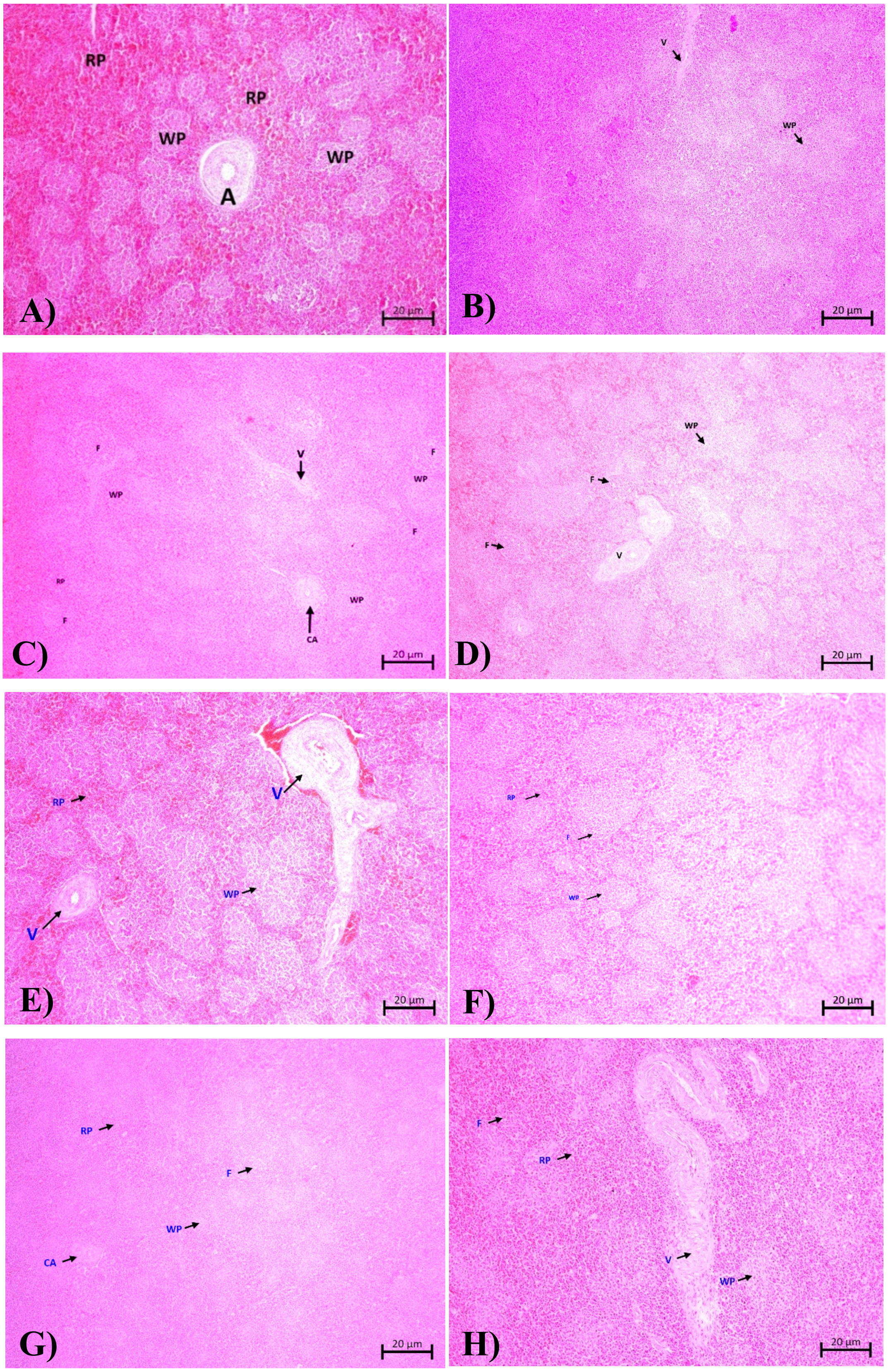
A)
RP
RP
WP
WP
A
20 µm
B)
V
WP
20 µm
C)
F
V
F
WP
WP
F
RP
F
WP
CA
20 µm
D)
WP
F
F
V
20 µm
E)
V
RP
WP
V
20 µm
F)
RP
F
WP
20 µm
G)
RP
F
WP
CA
20 µm
H)
F
RP
V
WP
20 µm

Figure S1. An overview of histopathological assessment in the broiler spleen experiment group in low magnification (20×, H&E Stain). **A:** Group C1; **B**: Group C2; **C**: Group C3; **D**: Group C4; **E**: Group C5; **F**: Group C6; **G**: Group C7; **H**: Group C8. (**A)** The negative control group exhibited a compact spleen architecture, making it easy to differentiate between WP, RP, and blood vessels. **(B)** In the positive control group, the *E. coli* infection damaged the vessel wall, resulting in vasculitis (V), which was indistinguishable from WP and RP. **(C)** Group experiment C3 shows several vasculitides (V) with a distinct WP. The splenic follicles (F) are dense enough to make a clear marginal zone from the RP area. **(D**) Group experiment C4 with a higher dose of JCEO shows a more contrast zone between RP and WP, while vasculitis remains intact. **(E)** The single-dose group of JCEO, C5, exhibits severe damage to the blood vessel walls, with a thickened fibrin matrix serving as a substitute for damaged endothelial cells, resulting in a narrowed vessel lumen. Yet, the inner structure remains visible to identify. **(F)** The C6 group exhibits a clear separation between WP and RP, with some follicles densely populated by lymphocytes. **(G)** C7 portrays the same coalescent structure as the C2 group; the cell degeneration is stained pale white, making the follicle loose. **(H)** The C8 group exhibits moderate vasculitis; no congestion is apparent, and WP are easily identifiable, while some lymphoid cells inside are observed as necrotic.

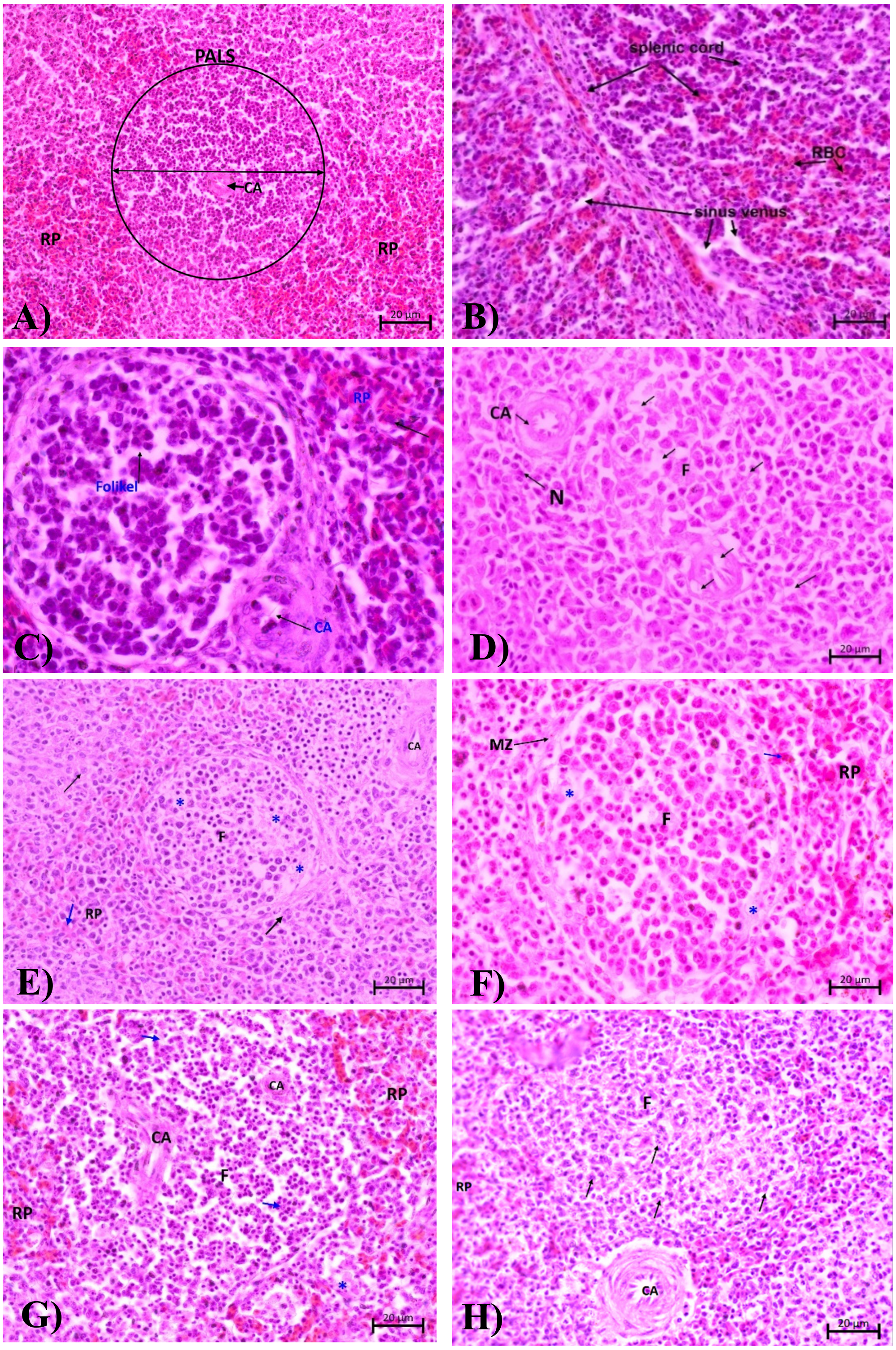
A)
PALS
CA
RP
RP
20 µm
B)
splenic cord
RBC
sinus venus
C)
RP
Folikel
CA
D)
CA
N
F
20 µm
E)
CA
F
RP
20 µm
F)
MZ
RP
F
20 µm
G)
CA
RP
CA
F
RP
20 µm
H)
F
RP
CA
20 µm

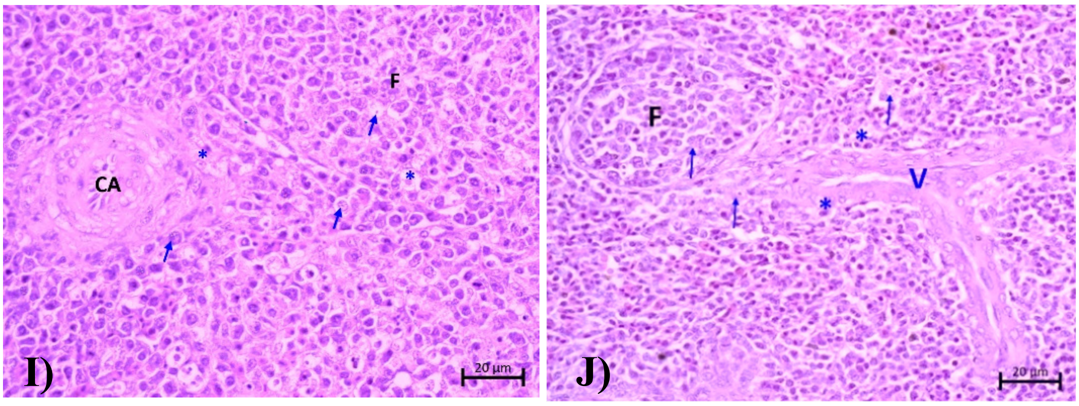

Figure S2. Spleen WP and RP architecture damage across the groups in high magnification (400×, H&E Stain). A – C : Group C1; D: Group C2; E: Group C3; F: Group C4; G: Group C5; H: Group C6; I: Group C7; J: Group C8. (A – C) The C1 group depicts a detailed structure in WP (S2A and S2C) and RP (S2B). WP consists of two components, the PALS zone and the splenic follicle (F). PALS consists of mature T cell lymphocytes surrounding the central arteriole (CA). Splenic follicles consist of indifferent and immature lymphocytes with CA near the structure. The follicles are surrounded by the splenic cord of Billroth, which extends into the RP zone (S2B), also known as the marginal zone. The Cord of Billroth is a loose network of reticular fibres and reticular cells that support macrophages, plasma cells, blood cells, and hematopoietic cells. Inside the RP zone are dense erythrocytes that flow through the venous sinuses. (D) C2 shows a depletion of lymphoid follicle (F), the lymphocytes undergo degeneration (black arrows), appearing cloudy, and others experience a karyolysis necrosis (N). (E-F) degeneration cells with abnormal cytoplasm structure (blue asterisk) and an abundance of nuclear debris in one cell (blue arrows). The marginal zone (MZ) remains visible, separating the follicles and RP zone. (G-I) A disintegrated structure of splenic follicles, a bunch of ghost cells creating a cloudy-like appearance (blue asterisks), and nuclei were karyohectic (black arrows). (J) The C8 antibiotic group indicates several lymphocytes with necrotic cells (blue arrows) and vasculitis, but follicles remain intact.

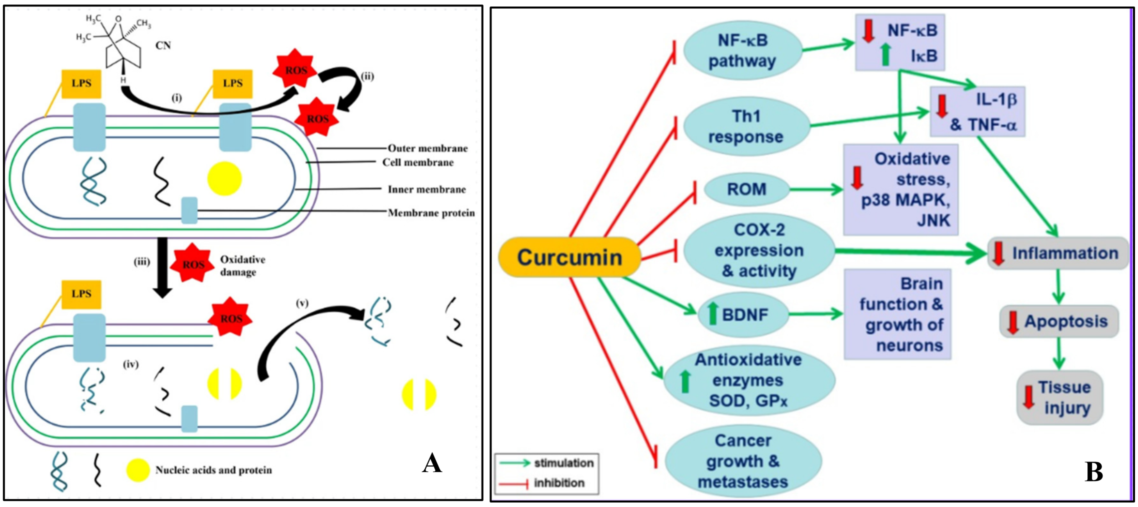

Figure S3. The mechanism of cineol against bacteria (A) and curcumin's properties (B) towards immune responses. (A) The 1,8-cineol bactericidal mechanism against bacteria. Cineol interrupts the porin

membrane located in the LPS of the outer layer. Then, it induced ROS species, leading to bacterial cell death [37]. (B) Curcumin intervenes in several inflammatory pathways inside the body, resulting in minimal tissue damage [43].

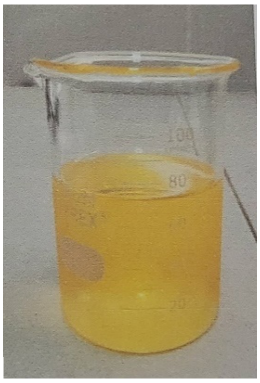
A)

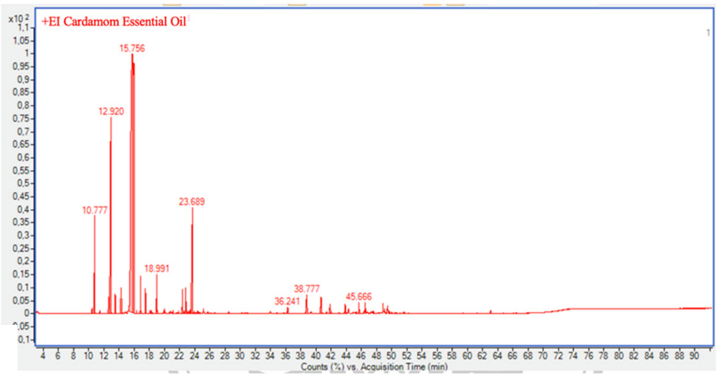

B)

Figure S4. Physical properties and gas chromatography-mass spectrometry result of Javanese cardamom essential oil (JCEO)

A) Physical characteristic of JCEO include:

a. Sample mass: 21,78 kg;
b. Colour and odor: yellow transparent, unscented;
c. Density: 0.98 g/mL.

B) Gas-chromatography-mass spectrometry results indicated the dominant component detected is Eucalyptol with 39.05% area within 15.7-minute retention time. Followed by 20.92% eucalyptol in 15.82 minutes of retention time and bicyclo[3.1.1]heptane,6,6-dimethyl-2-methylene (β-Pinene) with 11.72% area and 12.9 retention time.

A) 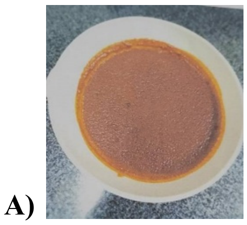

B) 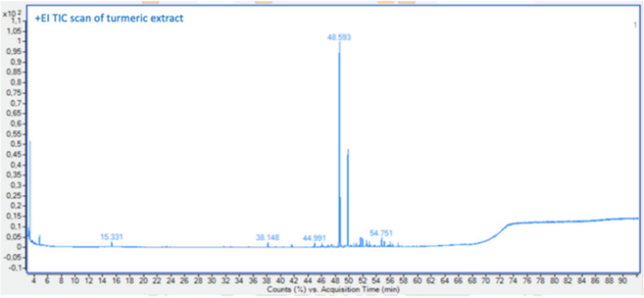

Secondary Metabolic	Reagent	Extraction Result	Interpretaion[1]
Phenolic	5% $FeCl_3$	Deep green	+++
Tannin	1% $FeCl_3$	Deep green	+++
Flavonoid	Concentrated HCL +Mg<br>H_2SO_4 2N<br>NaOH 10%	Orange-reddish<br>Orange<br>Pale brown	+<br>+<br>+
Triterpenoid and Steroid	Heating process concentrated H_2SO_2 reactant+ CH_3COOH anhidrat	Yellow, with a clumpy texture	+++
Alkaloid	Dragendorff reactant	Red	-

C) [1](+): less amount , (++): moderate amount, (+++): numerous amount of, (-): not available/not detected.

Figure S5. Physical Properties, phytochemical test, and Gas chromatography-mass spectrometry result of dried turmeric ethanol extract (DT):

A) Physical characteristic of dried turmeric ethanol extracts include:

a. Extraction mass: 20,17 grams;
b. Colour: saturated yellow;

B) Gas-chromatography-mass spectrometry results indicated the dominant component detected is aR-Turmerone with 46.6% area within 48.59-minute retention time. Followed by 18.16% of Curlone for 49.82 minutes of retention time and Turmerone with 10.08% area and 48.84 retention time.

C) Phytochemical substances extracted are phenol, tannin, triterpenoid and steroid, and flavonoid.

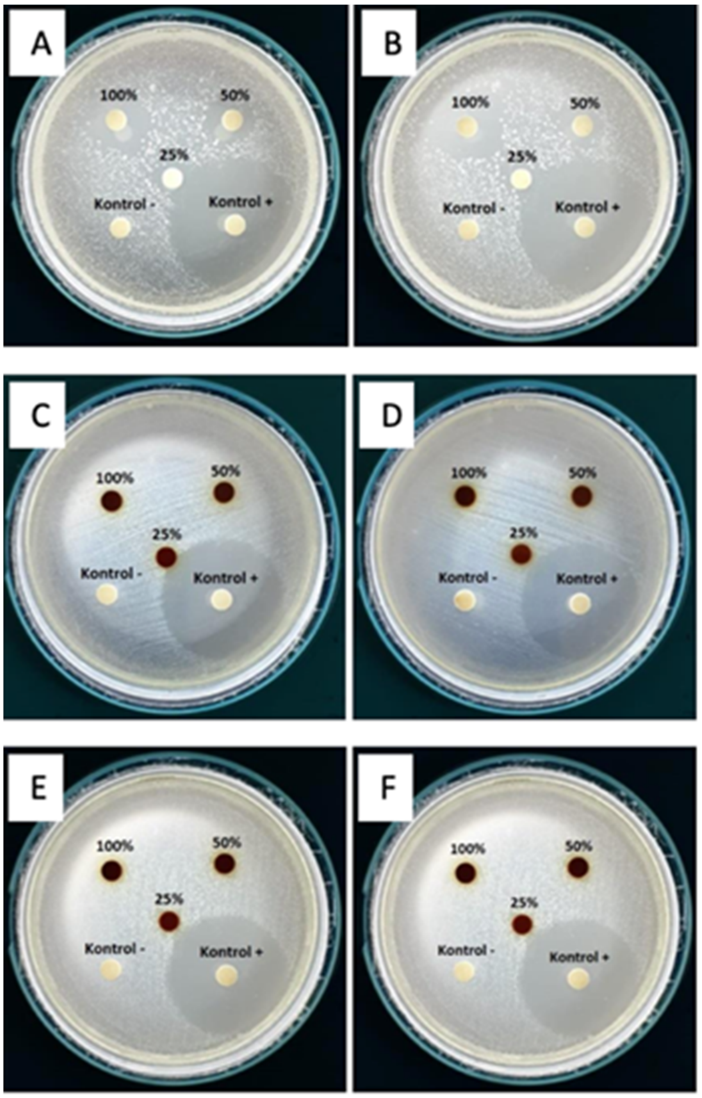

Figure S6. The antibacterial activity of extracts against the *E. coli* O78 strain. The result presents the key findings on the antibacterial activity of JCEO and DT extracts against the *E. coli* O78 strain. The figures clearly show the formation of a clear zone around the JCEO discs in varying concentrations (25%, 50%, and 100%) (A-B), indicating their antibacterial activity. Similarly, the DT discs in varied concentrations also show antibacterial activity (C-D). The combination of JCEO and DT in various concentrations also exhibits antibacterial activity (E-F).

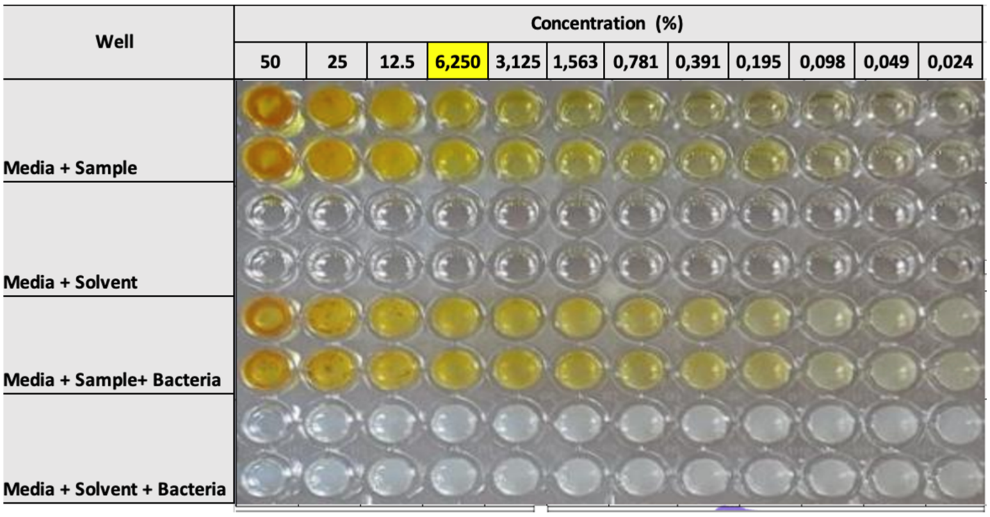

Figure S7. MIC of JCEO: DT combination (1:1) against *E. coli* O78 at a minimum concentration of 6.250 % measured by the optical density of the dilutions incubated with the *E. coli*.

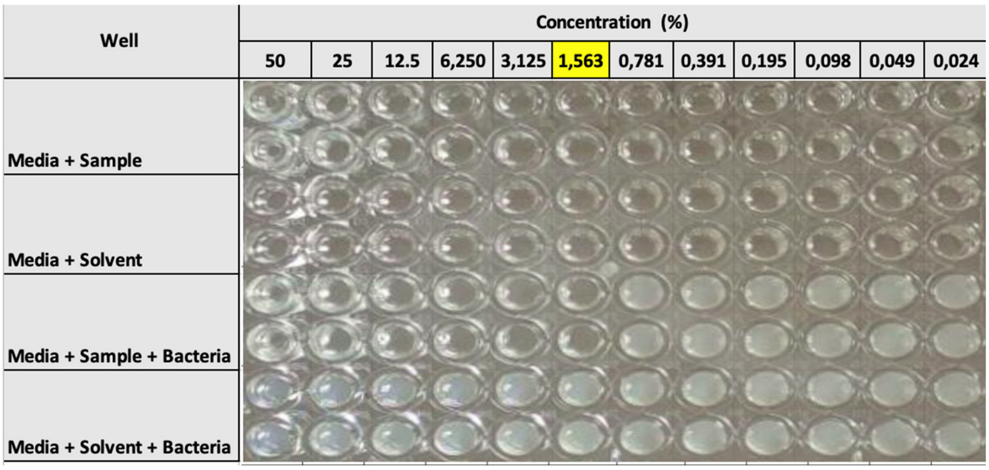

Figure S8. MIC of JCEO against *E. coli* O78 was measured by the optical density of the dilutions incubated with the *E. coli*.

References (The order is following the main text)

37. Hafez, M.H.; El-Kazaz, S.E.; Alharthi, B.; Ghamry, H.I.; Alshehri, M.A.; Sayed, S.; Shukry, M.; El-Sayed, Y.S. The Impact of Curcumin on Growth Performance, Growth-Related Gene Expression, Oxidative Stress, and Immunological Biomarkers in Broiler Chickens at Different Stocking Densities. *Animals* **2022**, 12, 958, doi:10.3390/ani12080958.
43. Kwiecien, S.; Magierowski, M.; Majka, J.; Ptak-Belowska, A.; Wojcik, D.; Sliwowski, Z.; Magierowska, K.; Brzozowski, T. Curcumin: A Potent Protectant against Esophageal and Gastric Disorders. *International Journal of Molecular Sciences* **2019**, 20, 1477, doi:10.3390/ijms20061477.
44. Santos FA, Silva RM, Campos AR, De Araujo RP, Júnior RL, Rao VS. 1,8- cineole (eucalyptol), a monoterpene oxide attenuates the colonic damage in rats on acute TNBS-colitis. *Food and Chemical Toxicology*. **2004** Apr 1;42(4):579-84.
